# Untreated Opioid Use Disorder and Health-Related Quality of Life Among Syringe Service Program Clients

**DOI:** 10.1001/jamanetworkopen.2024.5968

**Published:** 2024-04-05

**Authors:** Megan E. Deaner, Johnathan Rausch, Parker Entrup, O. Trent Hall

**Affiliations:** 1 Department of Psychiatry and Behavioral Health, The Ohio State University Wexner Medical Center, Columbus; 2 College of Medicine, The Ohio State University, Columbus

## Abstract

This cross-sectional study explores health-related quality of life (HRQoL) and the challenges to physical, emotional, and social functioning among individuals with opioid use disorder.

## Introduction

The mortality burden of opioid use disorder (OUD) in the United States is widely reported.^1^ Less understood is the effect of untreated OUD on health-related quality of life (HRQoL). The World Health Organization defines quality of life as “an individual’s perception of their position in life in the context of the culture and value systems in which they live and in relation to their goals, expectations, standards, and concerns.”^2^ HRQoL refers to perceptions of mental, physical, and social well-being; and how health affects functional ability. Understanding HRQoL is important for improving symptom relief, care, and rehabilitation in chronic health conditions.^3,4^ Understanding the association between OUD and HRQoL may be crucial for developing comprehensive and person-centered approaches to prevention, treatment, and recovery support. This study explores HRQoL and the challenges to physical, emotional, and social functioning faced by individuals with OUD.

## Methods

This survey-based cross-sectional study followed the STROBE reporting guideline. Approval was granted by The Ohio State University Wexner Medical Center institutional review board. Participants provided verbal consent and were adults (recruited from a syringe service program in Columbus, Ohio, from January 10 to February 21, 2023) who agreed they were “not seeking or engaged in any substance use treatment.” This single electronic survey assessed demographic variables, sample characteristics of opioid use, *Diagnostic and Statistical Manual of Mental Disorders, Fifth Edition (DSM-5)* OUD criteria, and HRQoL as measured by the RAND Corporation 36-Item Health Survey 1.0 (RAND-36).^5^ RAND-36 consists of 8 HRQoL domains: general health, physical functioning, mental health, social functioning, vitality, bodily pain, role limitations due to physical health, and role limitations due to emotional problems. A domain score of 100 indicates optimal life quality. This study only included individuals who completed RAND-36 (n = 97). Racial identity and gender were self-reported. Race was assessed to provide a comprehensive demographic profile, which contextualizes findings and is crucial for gauging generalizability or limitations therein. SPSS Statistics version 28.0 (IBM Corp) was used to analyze data from August 1 to November 15, 2023. See the eAppendix in Supplement 1 for additional methods details.

## Results

Among 97 participants included in the study sample, 53 (55.2%) were male; 11 (11.3%) were Black (only), 79 (81.4%) were White (only), and 7 (7.2%) were other race or multiracial; 86 (88.7%) were non-Hispanic; and mean (SD) age was 37.1 (8.0) years (Table). Of the 97 participants, 88 (90.7%) had used opioids in the past week. However, all 97 met criteria for active OUD (≥2 *DSM-5* OUD criteria); and 92 (94.8%) met criteria for severe OUD (≥6 *DSM-5* criteria). For 7 of 8 RAND-36 life domains, median scores were less than half the optimum. The lowest scores were seen in role limitations due to mental health, which had lower scores than social functioning and mental health. The Figure summarizes results per domain.

**Table.** Sample Characteristics (N = 97)^a^

Characteristic	No. (valid %)
Age, mean (SD), y	37.1 (8.0)
Gender	
Cisgender female	40 (41.7)
Cisgender male	53 (55.2)
Prefer not to say	3
Racial identity	
Black (only)	11 (11.3)
White (only)	79 (81.4)
Other or multiracial^b^	7 (7.2)
Ethnicity	
Hispanic	1 (<0.1)
Non-Hispanic	86 (88.7)
Prefer not to say	9 (9.3)
OUD severity, median (IQR)	
*DSM-5 *criteria	11 (10-11)
Route of administration, No. reports	
Intravenous	72
Intramuscular or subcutaneous	7
Smoking	41
Snorting/sniffing up the nose	19
Swallowing by mouth	5
Another route	1
Monetary cost of daily opioid use, median (IQR), $	60 (30-100)

Abbreviations: *DSM-5*, *Diagnostic and Statistical Manual of Mental Disorders, Fifth Edition*; OUD, opioid use disorder.

^a^ All information in the table, including demographics and OUD *DSM-5* criteria, was entirely self-reported and not determined by the investigators or clinicians. Eleven participants were missing data for monetary cost of daily opioid use. One participant was missing data for age. Gender was missing 3 entries. Ethnicity was missing 1 entry.

^b^ The other and multiracial category includes all individuals who selected more than 1 racial identity from the available options (Asian, Black, Native American or Alaska Native, Native Hawaiian or Other Pacific Islander, White).

**Figure.**
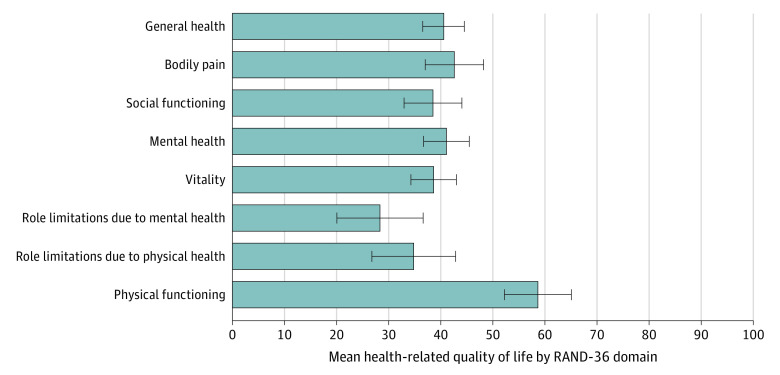
Mean Health-Related Quality of Life by RAND 36-Item Health Survey 1.0 Health-related quality of life among non–treatment-seeking individuals with opioid use disorder across 8 domains. A score of 100 represents optimal quality of life. Error bars indicate SD.

## Discussion

This study found that OUD was associated with HRQoL deficits across multiple domains, with the worst outcomes seen in role limitations due to mental health. Median scores were higher in the mental health and social functioning domains. Role limitations due to physical health was also associated with lower quality of life. Physical complications, as well as the cycle of opioid withdrawal and relapse, can substantially reduce an individual’s quality of life, contributing to the extreme frustrations and challenges experienced by many with this chronic health condition. Understanding HRQoL in OUD could improve treatment engagement and outcomes.

People with OUD also experience stigma from a variety of sources, warranting further research on its intersection with HRQoL.^6^ HRQoL of people with OUD may be improved by collaboration between researchers, health care clinicians, policy makers, and community organizations. Limitations of this study include a cross-sectional design and limits on generalizability; individuals using syringe program services may have differences to the broader population, the study took place at 1 location, and there is difficulty in accessing a large sample of individuals with untreated OUD. Nevertheless, this study underscores a hidden crisis in health and quality of life among those with OUD, emphasizing the need to prioritize HRQoL in harm reduction and treatment settings.

## Notes

Supplement 1. **eAppendix.** Supplemental Methods
eReferences

Supplement 2.
Data Sharing Statement
