# Supplementary material for: Untreated Opioid Use Disorder and Health-Related Quality of Life Among Syringe Service Program Clients
Source: JAMA Netw Open. 2024 Apr 5;7(4):e245968. doi: 10.1001/jamanetworkopen.2024.5968 (PMC10998153; doi:10.1001/jamanetworkopen.2024.5968)
Supplement: Supplement 1. — eAppendix. Supplemental Methods eReferences [file jamanetwopen-e245968-s001.pdf]

Supplemental Online Content

Deaner ME, Rausch J, Entrup P, Hall OT. Untreated opioid use disorder and health-related quality of life among syringe service program clients. *JAMA Netw Open*. 2024;7(4):e245968. doi:10.1001/jamanetworkopen.2024.5968

eAppendix. Supplemental Methods

eReferences

This supplemental material has been provided by the authors to give readers additional information about their work.

© 2024 Deaner ME et al. *JAMA Network Open.*

eAppendix. Supplemental Methods

Electronic surveys were administered via tablet computers using REDCap. Redcap is a secure platform for storing personal health information in a manner that meets HIPPA compliance standards.[1] The study included syringe service program participants 18 years of age or older who reported agreement that they were "not seeking or engaged in any substance use treatment." Substance use treatment was left open to participant interpretation, and no attempt was made to explicitly differentiate engagement with pharmacological vs non-pharmacological treatment modalities. This was done to assess a population that does not consider themselves as treatment-engaged in any capacity. Exclusion criteria included the inability to provide informed consent, read, comprehend survey questions, or reported that they were engaged in substance use treatment. Inclusion and exclusion criteria were screened and confirmed verbally prior to the electronic survey. The study assessed OUD as defined by the Diagnostic and Statistical Manual of Mental Disorders, Fifth Edition (DSM-5).[2]Participants self-reported these criteria using a questionnaire created by the National Institute on Drug Abuse (NIDA).[2]

HRQoL was measured with the Research and Development (RAND) Corporation RAND 36-Item Health Survey 1.0 (RAND-36).[3] RAND-36 is a license-free equivalent to the 36-item short-form questionnaire (SF-36) and is openly available from the RAND Corporation.[4] RAND-36 has been repeatedly and well-validated for assessing health-related quality of life[3,5–7]. Scoring of RAND-36 involves responding to multiple-choice questions or ranking statements with a mix of Likert and dichotomous response options. Scoring RAND-36 involves the linear transformation of its 36 questions such that all scores are taken out of 100 (i.e., transformed to represent a

© 2024 Deaner ME et al. *JAMA Network Open.*

percentage of the maximum possible score), followed by averaging of items that fall under the same domain to generate a final score under each of the 8 domains.[3,8–11]

eReferences

1. Harris PA, Taylor R, Thielke R, Payne J, Gonzalez N, Conde JG. Research electronic data capture (REDCap)—a metadata-driven methodology and workflow process for providing translational research informatics support. *Journal of biomedical informatics*. 2009;42(2):377-381.

2. National Institute on Drug Abuse. Questions for Identification of Opioid Use Disorder Based on DSM-5. NIDAMED: Medical & Health Professionals. https://nida.nih.gov/nidamed-medical-health-professionals/your-discipline/emergency-physicians-first-responders/questions-identification-opioid-use-disorder-based-dsm-5. Accessed 2023.

3. Hays RD, Morales LS. The RAND-36 measure of health-related quality of life. *Annals of medicine*. 2001;33(5):350-357.

4. RAND Corporation. The MOS 36-Item Short-Form Health Survey (SF-36). RAND Health Care. Accessed [February 7th, 2024]. https://www.rand.org/content/dam/rand/www/external/health/surveys_tools/mos/mos_core_36item_survey.pdf.

5. Lins-Kusterer L, Aguiar I, Santos-Lins LS, et al. Validation of the RAND 36-Item Health Survey questionnaire in Brazil. *Arquivos de Gastroenterologia*. 2022;59:193-197.

6. de Vries CE, Makarawung DJ, Monpellier VM, Janssen IM, de Castro SM, van Veen RN. Is the RAND-36 an adequate patient-reported outcome measure to assess health-related quality of life in patients undergoing bariatric surgery? *Obesity Surgery*. Published online 2022:1-7.

7. Kaarlola A, Pettilä V, Kekki P. Performance of two measures of general health-related quality of life, the EQ-5D and the RAND-36 among critically ill patients. *Intensive care medicine*. 2004;30:2245-2252.

8. Coons SJ, Alabdulmohsin SA, Draugalis JR, Hays RD. Reliability of an Arabic version of the RAND-36 Health Survey and its equivalence to the US-English version. *Medical care*. Published online 1998:428-432.

9. Hays R. The RAND-36 measure of health-related quality of life. *The RAND-36 measure of health-related quality of life*. doi:10.3109/07853890109002089

10. Ware Jr JE, Sherbourne CD. The MOS 36-item short-form health survey (SF-36): I. Conceptual framework and item selection. *Medical care*. Published online 1992:473-483.

11. Hays RD, Shapiro MF. An overview of generic health-related quality of life measures for HIV research. *Quality of life research*. 1992;1(2):91-97.

© 2024 Deaner ME et al. *JAMA Network Open.*
