# Supplementary material for: Untreated Opioid Use Disorder and Health-Related Quality of Life Among Syringe Service Program Clients
Source: JAMA Netw Open. 2024 Apr 5;7(4):e245968. doi: 10.1001/jamanetworkopen.2024.5968 (PMC10998153; doi:10.1001/jamanetworkopen.2024.5968)
Supplement: Supplement 2. — Data Sharing Statement [file jamanetwopen-e245968-s002.pdf]

Data Sharing Statement

Deaner. Untreated Opioid Use Disorder and Health-Related Quality of Life Among Syringe Service Program Clients. *JAMA Netw Open*. Published April 05, 2024. doi:10.1001/jamanetworkopen.2024.5968

Data
Data available: No
